# Long-term safety of filgrastim (rhG-CSF) administration

**DOI:** 10.1111/j.1365-2141.2007.06524.x

**Published:** 2007-04

**Authors:** Dennis L Confer, John P Miller

**Affiliations:** National Marrow Donor Program®, 3001 Broadway St NE, Suite 500 Minneapolis, MN, USA. Email: dconfer@nmdp.org

**Keywords:** colony-stimulating factor, haematological malignancy

This journal recently included an article by Bennett *et al* (2006) reporting five cases of haematological malignancy occurring in normal individuals following exposure to haematopoietic growth factors. Three cases described lymphoid malignancies (non-Hodgkin lymphoma and chronic lymphocytic leukaemia) occurring months to years after receipt of an investigational agent (pegylated, recombinant megakaryocyte growth and development factor). The two additional cases, however, were identified among 200 individuals who had received filgrastim (recombinant human granulocyte colony-stimulating factor; rhG-CSF, Neupogen®) as a mobilising agent for collection of peripheral blood stem cells (PBSC) for allogeneic transplantation. The transplant recipients for these latter two donors were their siblings, each with acute myelogenous leukaemia (AML). The donors themselves also developed AML 4–5 years following filgrastim exposure.

It is known that siblings of persons with leukaemia have a 2–5-fold increased annual incidence of leukaemia (Pottern *et al*, 1991; Shpilberg *et al*, 1994; Rauscher *et al*, 2002). In some families, multi-generational occurrence of leukaemia, in the absence of any known syndrome, e.g. Fanconi anaemia, suggests direct inheritance of susceptibility genes (Segel & Lichtman, 2004). Given these patterns, the contribution of filgrastim exposure to the development of acute leukaemia within families remains speculative.

Documenting the safety of filgrastim as a mobilising agent for PBSC donation has long been a matter of importance for the transplantation community, particularly in the context of donations made by volunteer, unrelated adult donors. Since 1997, the US National Marrow Donor Program (NMDP) has maintained an Investigational New Drug (IND) application accepted by the Food and Drug Administration for manufacture of PBSC products from unrelated donors. Filgrastim is administered for PBSC mobilisation at a total dose of *c.* 10 μg/kg donor weight per day for 5 d. Under the IND protocols, every donor provides informed consent for the research, which includes agreement for perpetual annual follow-up. Among 4015 donors who have passed the first anniversary of their PBSC donation, we have accumulated 9785 years of follow-up (range 1–9 years with 897 donors ≥4 years). Twenty cases of cancer have been reported, occurring in various organ systems, consistent with the age-adjusted US incidence of cancer in adults and in support of the applicability of data obtained from the NMDP follow-up system (Ries *et al*, 2006). There have been no reports of leukaemia or lymphoma in this donor cohort, which US statistics suggest should comprise 9% of all malignancies in this age group.

National Marrow Donor Program donor consent forms approved by the Institutional Review Board contain the following information:

Normal individuals are at risk for developing cancer, including leukaemia, lymphoma or other blood diseases throughout their lifetime. It is unknown whether filgrastim increases or decreases an individual's risk of developing cancer. The data being collected during follow-up will help establish if there are any positive or negative long-term effects from receiving filgrastim. Based on limited long-term data from healthy people who have received filgrastim, no long-term risks have been found so far.

The low occurrence of leukaemia and lymphoma in our cohort of volunteer, unrelated PBSC donors should provide reassurance to individuals who receive filgrastim for PBSC mobilisation and should encourage their participation in carefully designed programmes for follow-up monitoring. As data from these and other studies mature, a more complete assessment of overall donor safety will become available to all interested parties.
